# Spatial inequalities in quality antenatal care in India: a district-level analysis

**DOI:** 10.3389/fpubh.2026.1878310

**Published:** 2026-07-17

**Authors:** Rahul Nandan, Ashutosh Kumar, Uttara Singh, Azizur Rahman Siddiqui, Anurag Yadav

**Affiliations:** 1 University of Allahabad CMP Degree College, Prayagraj, India; 2 Govind Ballabh Pant Social Science Institute, Prayagraj, India; 3 University of Allahabad, Prayagraj, India

**Keywords:** geographically weighted regression, maternal healthcare utilization, spatial analysis, spatial error model, spatial lag model

## Abstract

The study examines the spatial distribution and determinants of quality antenatal care (Q-ANC) utilization across districts of India using data from the National Family Health Survey (NFHS-5, 2019–21). Q-ANC is operationalized as the receipt of at least five out of seven essential antenatal care components, capturing the adequacy of service content during pregnancy. District-level estimates were analyzed using spatial statistical techniques, including Global and Local Moran’s I, spatial regression models, and geographically weighted regression (GWR), to identify clustering patterns and spatial heterogeneity in determinants. The findings reveal substantial regional disparities in Q-ANC utilization, with higher coverage concentrated in southern India and lower levels in northern and northeastern districts. Significant positive spatial autocorrelation (Moran’s I ≈ 0.60, *p* < 0.001) indicates strong geographic clustering. Spatial regression results show that intended pregnancy, adverse pregnancy outcomes, mass media exposure, and utilization of public healthcare facilities are positively associated with Q-ANC, whereas higher parity and greater concentration of socially disadvantaged and minority populations are negatively associated. The GWR model demonstrates considerable spatial variation in these relationships, explaining up to 80% of the observed variation across districts. These findings highlight that the determinants of maternal healthcare utilization are not spatially uniform and are shaped by localized socio-demographic and health system factors. The study underscores the need for geographically targeted policy interventions to address persistent regional inequalities in maternal healthcare access and quality in India.

## Background

Maternal health continues to remain a critical public health priority, particularly in the context of the Sustainable Development Goals (SDGs), which aim to reduce the global maternal mortality ratio (MMR) to fewer than 70 deaths per 100,000 live births by 2030 (1). Over the past two decades, substantial progress has been achieved, with global estimates from the World Health Organization and UNICEF indicating a decline of nearly 40% in maternal mortality between 2000 and 2020 (2, 3). Despite these improvements, maternal mortality remains highly uneven across regions, with low- and middle-income countries (LMICs) continuing to bear a disproportionate burden. Countries such as India still report relatively higher maternal mortality levels (approximately 87 deaths per 100,000 live births), compared to high-income countries where the ratio typically ranges between 10 and 20 deaths per 100,000 live births (4, 5). This persistent disparity reflects not only differences in healthcare access but also variations in the quality and effectiveness of maternal healthcare services. Evidence suggests that timely and appropriate care during pregnancy, childbirth, and the postnatal period plays a crucial role in reducing maternal and neonatal morbidity and mortality (6). Within this continuum of care, antenatal care (ANC) services serve as a critical entry point for early detection of complications, risk management, and strengthening linkages between women and the health system (7).

Despite the overall decline in maternal mortality, substantial interstate disparities continue to persist across India (8). According to the latest Sample Registration System (SRS) Maternal Mortality Bulletin (2022–24), the national maternal mortality ratio stands at 87 deaths per 100,000 live births; however, considerable regional variation is evident. The Empowered Action Group (EAG) states and Assam report a combined MMR of 116, compared with only 41 in the southern states. Furthermore, states such as Uttar Pradesh (154), Madhya Pradesh (135), Chhattisgarh (124), and Odisha (124) continue to experience substantially higher maternal mortality, whereas Kerala (24) and Tamil Nadu (25) have achieved markedly lower levels (5). These pronounced regional differences reflect persistent inequalities in healthcare access, service utilization, health system performance, and socio-economic development (9, 10). Understanding the spatial distribution of maternal healthcare utilization is therefore critical for identifying geographically disadvantaged areas and designing targeted interventions to improve maternal health outcomes.

In the Indian context, improving maternal healthcare utilization has been a major policy priority through several large-scale initiatives aimed at strengthening maternal and child health services. The National Health Mission (NHM) and Janani Suraksha Yojana (JSY) have played a crucial role in expanding access to antenatal care, skilled birth attendance, and institutional delivery services, particularly among socio-economically disadvantaged populations (1). Recognizing that improved maternal health outcomes depend not only on service contact but also on the quality of care provided, the Government of India launched the Pradhan Mantri Surakshit Matritva Abhiyan (PMSMA) in 2016 to ensure the provision of comprehensive and quality antenatal care services through designated monthly ANC clinics at public health facilities. PMSMA emphasizes essential antenatal screening, risk identification, early detection of pregnancy-related complications, and timely referral of high-risk pregnancies (11). These initiatives have contributed to a steady increase in the coverage of antenatal care services across the country. For instance, data from the National Family Health Survey (NFHS-5) indicate that approximately 58.6% of women received four or more ANC visits during their most recent pregnancy (12). Nevertheless, national-level improvements conceal substantial geographic inequalities in maternal healthcare utilization. Recent district-level studies based on NFHS-5 data have documented marked interstate and district-level variation in both antenatal care utilization and quality antenatal care coverage, with districts in southern India consistently demonstrating better maternal healthcare performance, while several districts in Bihar, Uttar Pradesh, Jharkhand, and the northeastern region continue to experience comparatively lower service utilization and poorer maternal healthcare outcomes (12, 13). Beyond these geographic disparities, significant socio-economic and demographic inequalities also persist. Existing literature highlights that factor such as maternal education, household wealth, parity, exposure to mass media, and place of residence play a crucial role in shaping access to antenatal care services in India (14, 15). Moreover, regional differences in health infrastructure, governance capacity, and broader socio-economic development further contribute to uneven patterns of maternal healthcare utilization across states and districts (16, 17). These findings suggest that improvements in maternal health outcomes are not solely dependent on increasing service coverage, but also on addressing underlying structural and contextual inequalities.

While the number of antenatal care visits is commonly used as an indicator of service utilization, it does not adequately capture the quality or content of care received during pregnancy (18, 19). International guidelines emphasize that effective antenatal care should include a range of essential clinical services, such as blood pressure monitoring, blood and urine testing, tetanus toxoid immunization, and iron–folic acid supplementation (20). These components are increasingly being used in empirical research to assess the adequacy or quality of antenatal care. Evidence from nationally representative surveys suggests that the receipt of these essential services varies substantially across socio-economic groups and geographic regions in India (14, 21). This indicates that improvements in maternal healthcare require a shift in focus from mere contact with healthcare services to the completeness and quality of care delivered during pregnancy.

In recent years, the concept of quality antenatal care (Q-ANC) has gained increasing attention in the literature, emphasizing the importance of service content rather than only service utilization (19, 22, 23). Studies using large-scale datasets have demonstrated that the receipt of recommended ANC components varies widely across population groups, with socio-economically disadvantaged and marginalized communities often experiencing lower levels of service quality (21, 24). More recent evidence also suggests that disparities in the quality of antenatal care persist even in settings where overall service coverage has improved, particularly across rural–urban contexts and among socially disadvantaged populations (25, 26). Although these studies provide valuable insights into the determinants of Q-ANC utilization, much of the existing research relies on conventional regression-based approaches and is often limited to national or state-level analysis (27).

Such approaches may mask important geographic variations and localized clustering of maternal healthcare utilization, particularly in a country like India, which is characterized by substantial heterogeneity in socio-economic conditions, cultural practices, and health system capacity. The determinants of quality antenatal care are therefore likely to vary across smaller administrative units such as districts, where local contextual factors play a more prominent role. Spatial analytical approaches provide an important methodological advancement in this regard, as they allow for the identification of geographic clustering, spatial dependence, and location-specific variations in the determinants of healthcare utilization (28, 29). By incorporating spatial dimensions into the analysis, it becomes possible to move beyond average effects and better understand the uneven distribution of maternal healthcare services across regions.

In this context, the present study examines the spatial distribution of quality antenatal care utilization across districts of India and investigates the socio-economic and demographic determinants associated with it. Specifically, the study aims to (i) identify the spatial patterns and clustering of Q-ANC utilization across districts, and (ii) examine how selected socio-demographic and contextual factors influence its utilization in a spatial framework. Building on existing evidence on socio-economic inequalities in maternal healthcare, the study hypothesizes that Q-ANC utilization is not randomly distributed but exhibits significant spatial clustering, and that its determinants vary across regions due to differences in socio-economic conditions, health system characteristics, and access to healthcare resources.

## Data and methods

### Study design

The present study adopts an ecological cross-sectional design to examine the spatial distribution and determinants of quality antenatal care (Q-ANC) utilization across districts of India. The analysis is based on district-level indicators derived from the National Family Health Survey (NFHS-5, 2019–21), where individual-level information was aggregated to the district level. Given that the unit of analysis is the district rather than the individual woman, the study investigates contextual and spatial variations in Q-ANC utilization across geographic areas. The analytical framework integrates descriptive statistics with spatial econometric techniques to identify geographic clustering patterns and to assess the influence of socio-demographic and contextual factors on Q-ANC utilization within a spatially explicit framework.

### Data

The study utilizes data from the Individual Recode (IR) file of the fifth round of the National Family Health Survey (NFHS-5), conducted during 2019–21. NFHS-5 is a nationally representative household survey implemented by the International Institute for Population Sciences (IIPS) under the stewardship of the Ministry of Health and Family Welfare, Government of India. The survey provides comprehensive information on population health, maternal and child health, and healthcare utilization across all states and union territories of India.

The NFHS-5 follows a multistage stratified sampling design, ensuring representativeness at national, state, and district levels (30). Sampling weights were applied in the analysis to account for the complex survey design. For the present study, individual-level data were aggregated at the district level to generate contextual indicators of Q-ANC utilization and its determinants. It is important to note that the analysis is ecological in nature; Therefore, the findings reflect district-level associations and should not be interpreted as individual-level causal relationships.

### Outcome variable

The primary outcome variable is the utilization of quality antenatal care (Q-ANC). Moving beyond the conventional focus on the number of antenatal visits, the study operationalizes Q-ANC based on the content of care received during pregnancy. In line with the World Health Organization (WHO) recommendations on essential antenatal care components, seven service indicators available in the NFHS dataset were used: blood pressure measurement, blood sample testing, urine examination, tetanus toxoid vaccination, iron–folic acid supplementation, weight measurement, and counselling or related clinical check-ups during pregnancy (20, 30).

Each component was coded as a binary variable (1 = received, 0 = not received), and a composite index was constructed by summing across the seven indicators. These indicators represent essential components of antenatal care that collectively capture the quality of care received during pregnancy. Following the approach adopted in previous studies on quality antenatal care utilizing Demographic and Health Survey data, women who received at least five of the seven recommended ANC components were classified as having received quality antenatal care (27, 31). This threshold has been widely used in the literature to capture adequate receipt of essential antenatal care services while ensuring comparability across population groups. Subsequently, district-level Q-ANC utilization rates were computed by aggregating the proportion of women who met this criterion within each district.

### Predictor variables

The study incorporates a range of socio-demographic, household, and healthcare-related variables that have been widely associated with maternal healthcare utilization in previous studies. These include maternal age, educational attainment, household wealth status, parity, place of residence (urban/rural), exposure to mass media, health insurance coverage, utilization of public healthcare facilities, pregnancy intention (wanted vs. unintended), and history of adverse pregnancy outcomes (26, 27).

All predictor variables were operationalized as district-level proportions or mean values, derived from individual-level responses (Supplementary Table S3). These aggregated indicators capture the broader socio-economic and contextual environment influencing maternal healthcare utilization across districts.

### Statistical analysis

To examine the spatial distribution and determinants of Q-ANC utilization, a combination of descriptive and spatial econometric techniques was employed.

Descriptive statistics:

Descriptive statistics were used to summarize the distribution of Q-ANC utilization and explanatory variables. District-level estimates were computed to examine regional variations and to provide an initial understanding of spatial disparities in maternal healthcare utilization across India.

To visualize the geographic distribution of Q-ANC utilization, district-level maps were prepared using ArcGIS. Districts were classified into five categories (very low, low, moderate, high, and very high) using the Natural Breaks (Jenks) classification method, which identifies natural groupings and minimizes within-class variation while maximizing between-class differences. The resulting Q-ANC utilization categories were: very low (32.78–58.51%), low (58.52–75.38%), moderate (75.37–86.43%), high (86.44–94.12%), and very high (94.13–100.00%).

Spatial autocorrelation analysis (Global Moran’s I and LISA):

To assess the presence of spatial dependence in Q-ANC utilization, Global Moran’s I statistic was computed. A statistically significant Moran’s I indicate that the spatial distribution of Q-ANC utilization is not random but exhibits clustering patterns. Statistical significance was assessed based on *p*-values generated in GeoDa, with *p* < 0.05 considered statistically significant. Further, Local Indicators of Spatial Association (LISA) were employed to identify localized clusters, including high-high (hotspots) and low-low (cold spots) regions. The spatial weights matrix was constructed based on first-order queen contiguity, and row-standardization was applied to ensure comparability across districts (32).

Ordinary least squares (OLS) regression:

An ordinary least squares (OLS) regression model was initially estimated to examine the association between district-level socio-demographic factors and Q-ANC utilization. The OLS model serves as a baseline specification, assuming spatial independence among observations. Diagnostic tests, including residual spatial autocorrelation, were considered to evaluate the adequacy of the OLS model. The presence of spatial dependence in residuals indicates the need for spatial econometric modelling. Multicollinearity among explanatory variables was evaluated using the Variance Inflation Factor (VIF), with values below 5 indicating the absence of severe multicollinearity (33, 34).

Spatial regression models:

To account for spatial dependence and potential violation of OLS assumptions, spatial econometric models were employed (35). Specifically, two alternative model specifications were estimated. Spatial Lag Model (SLM) incorporates a spatially lagged dependent variable to capture spatial spillover effects, reflecting the influence of neighbouring districts’ Q-ANC utilization on a given district. Spatial Error Model (SEM) accounts for spatial autocorrelation in the error term, which may arise due to omitted variables with spatial structure or unobserved regional heterogeneity. Model selection was guided by comparative fit statistics, including Akaike Information Criterion (AIC) and log-likelihood values, as well as the magnitude and significance of spatial parameters (*ρ* and *λ*).

Geographically weighted regression (GWR):

To further capture spatial heterogeneity in the relationships between predictors and Q-ANC utilization, geographically weighted regression (GWR) was employed. Unlike global models, GWR allows regression coefficients to vary across geographic space, thereby providing location-specific estimates. An adaptive kernel bandwidth was selected using the Akaike Information Criterion corrected (AICc) to ensure optimal model fit. Prior to model estimation, a Monte Carlo test for spatial variability was conducted to assess the presence of non-stationarity in regression coefficients (36, 37). The results indicated significant spatial variability (*p* < 0.05) for most explanatory variables, justifying the application of the GWR model (Supplementary Table S1). However, variables such as educational attainment, urban residence, and media exposure exhibited spatial stationarity, suggesting relatively stable effects across regions. The GWR model outputs were used to map local parameter estimates and local *R*^2^ values, enabling the identification of spatial variation in both model performance and the strength of associations.

## Results

### Descriptive statistics of the study variables

The descriptive statistics of the study variables indicate that approximately 89.0% of women received quality antenatal care (Q-ANC) during their most recent pregnancy, with a relatively high standard deviation (±11.6), reflecting substantial variability across districts (Table 1). The mean age of respondents was 27.5 years (±1.4). Around 24.0% (±21.0) of the women resided in urban areas with nearly three-fourths (76.0%) population living in rural areas, indicating a predominantly rural background sample.

**Table 1:** Descriptive statistics of the study variables (*n* = 707).

Variable	Mean/percentage	Standard deviation
Quality ANC utilization (%)	89.0	11.6
Respondent age (years)	27.5	1.4
Education (secondary or above) (%)	71.1	16.9
High parity (%)	28.0	12.6
Residence (urban) (%)	24.0	21.0
Minority status (%)	25.8	28.6
Household head relation (head/spouse) (%)	50.7	13.7
Wealth quintile (rich) (%)	35.9	24.7
Caste (SC/ST) (%)	39.6	23.1
Exposed to media (%)	75.9	17.5
Health insurance coverage (%)	27.5	22.4
Used public health facility (%)	53.8	17.5
Pregnancy wanted (%)	92.8	4.8
Adverse pregnancy outcome (%)	14.7	6.4

Statistics are calculated from district-level aggregated indicators derived from NFHS-5 (2019–21). Values represent the distribution of study variables across 707 districts.

In terms of educational attainment, more than two-thirds (71.1% ± 16.9) of the respondents had completed secondary education or above. Approximately 28.0% (±12.6) of women reported higher parity (three or more children). With respect to social composition, about 39.6% (±23.1) of the respondents belonged to Scheduled Caste/Scheduled Tribe (SC/ST) communities, while 25.8% (±28.6) were from religious minority groups.

Regarding access and exposure-related factors, a substantial proportion of women (75.9% ± 17.5) reported exposure to mass media. Similarly, 53.8% (±17.5) of the respondents utilized public healthcare facilities for maternal care services. In addition, a large majority of pregnancies (92.8% ± 4.8) were reported as intended, whereas 14.7% (±6.4) of women experienced adverse pregnancy outcomes.

### Spatial distribution of Q-ANC

**Figure 1:**
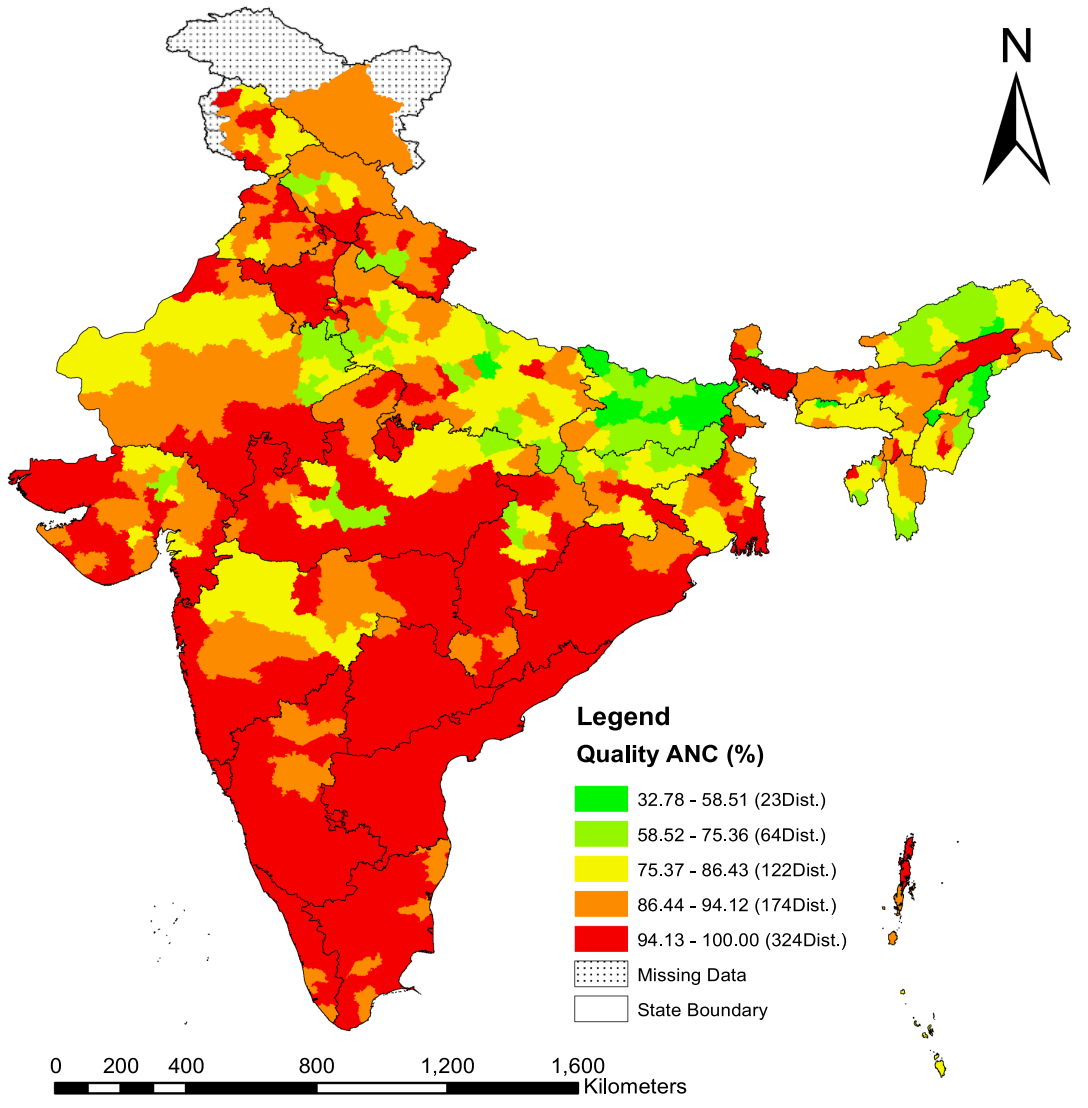
Geographical variation of quality antenatal care utilization across the India, NFHS-5 (2019–21). Districts were categorized into five classes ranging from very low to very high Q-ANC utilization based on natural breaks (Jenks) classification.

The spatial distribution of Q-ANC utilization reveals substantial geographic variation across districts of India. A clear regional pattern is observed, with districts located in the southern states Kerala, Karnataka, Tamil Nadu, Andhra Pradesh, Telangana, and Odisha exhibiting consistently high to very high levels of Q-ANC utilization, with coverage exceeding 85% in most districts (Figure 1).

In contrast, districts from Bihar and several states in northeastern India demonstrate comparatively very low to moderate levels of Q-ANC utilization, indicating persistent regional disparities. In the north western part of India, relatively higher utilization levels are observed in Haryana, along with selected districts of Himachal Pradesh and Uttarakhand.

A heterogeneous or mixed pattern of Q-ANC utilization is evident across the states of Uttar Pradesh and Madhya Pradesh, reflecting intra-state disparities. For instance, districts in southern Madhya Pradesh report higher utilization levels (often exceeding 94%), whereas northern parts of the state exhibit moderate levels of Q-ANC utilization. Similarly, within Uttar Pradesh, districts in the Bundelkhand region—including parts of western and eastern Uttar Pradesh show relatively higher utilization, while districts in the central region of the state demonstrate low to moderate levels.

### Spatial autocorrelation

**Figure 2:**
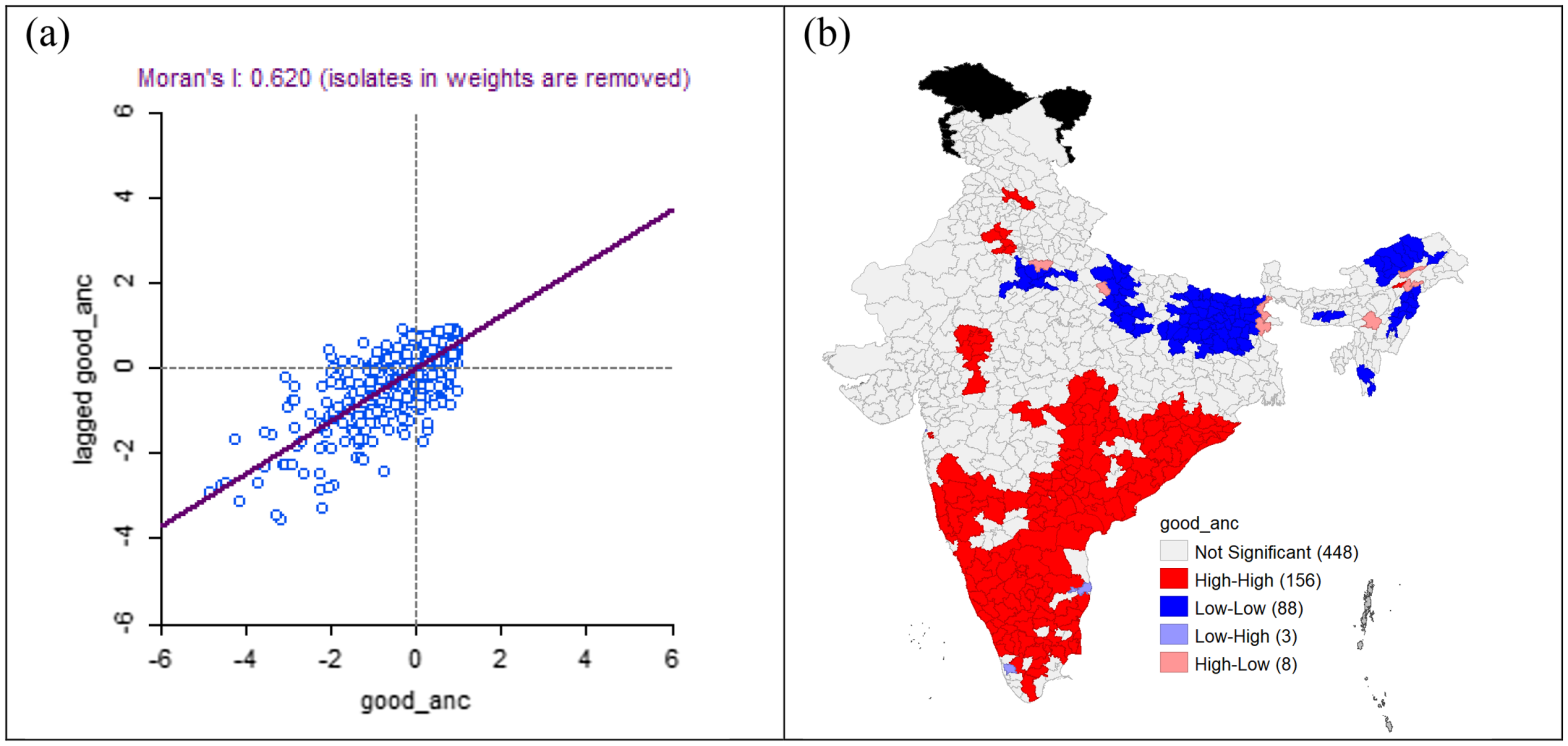
Spatial autocorrelation of quality antenatal care (Q-ANC) utilization across districts of India: **(a)** Global Moran’s I scatterplot and **(b)** LISA cluster map (*p* < 0.05). LISA clusters are significant at *p* < 0.05. Black areas indicate unavailable or missing data.

The results of Global Moran’s I indicate a statistically significant positive spatial autocorrelation in the distribution of Q-ANC utilization across districts of India (Moran’s I = 0.62, *p* < 0.001), suggesting that the pattern of utilization is not random but spatially clustered (Figure 2a). This implies that districts with high (or low) levels of Q-ANC utilization tend to be geographically proximate, reflecting underlying spatial dependence.

The Local Indicators of Spatial Association (LISA) further reveal distinct clustering patterns across the study area (Figure 2b). A total of 156 districts were identified as hotspot regions (high-high clusters), indicating significantly higher Q-ANC utilization surrounded by districts with similarly high levels. These clusters are predominantly concentrated in the southern states, including Kerala, Karnataka, Tamil Nadu, Andhra Pradesh, Telangana, and Odisha. In addition, a few localized hotspot clusters are observed in parts of central and northern India.

In contrast, 88 districts were identified as cold spot regions (low-low clusters), representing areas with persistently low Q-ANC utilization surrounded by similarly low-performing districts. These cold spots are primarily concentrated in Bihar, Uttar Pradesh, Arunachal Pradesh, and Nagaland, with smaller pockets observed in Meghalaya, Mizoram, and western Uttar Pradesh. The presence of such statistically significant clusters highlights pronounced spatial inequalities in maternal healthcare utilization across regions.

### Determinants of Q-ANC: global models

The results from the ordinary least squares (OLS) regression model indicate that the selected explanatory variables explain a substantial proportion of variation in district-level Q-ANC utilization, with an adjusted *R*^2^ value of 0.61. This suggests that socio-demographic and healthcare-related factors play a significant role in shaping the observed spatial variation. Additionally, the Variance Inflation Factor (VIF) values for all explanatory variables were found to be below the critical threshold of 5, indicating the absence of severe multicollinearity and confirming the robustness of the model estimates (Supplementary Table S2).

The analysis reveals that an increase in the proportion of women with secondary or higher education is associated with a marginal decline in Q-ANC utilization (*β* = −0.06). However, this association loses statistical significance in both the spatial lag and spatial error models (Table 2). This attenuation suggests that the initial OLS relationship may partly reflect spatially clustered socio-economic and contextual characteristics rather than an independent district-level effect of educational attainment. Consequently, the observed association should be interpreted with caution and within the broader spatial context of maternal healthcare utilization.

**Table 2:** Comparison of different models to evaluate the association between Q-ANC utilization rate across different socio-economic conditions, NFHS-5 (2019–21).

Variables	OLS (base model)	SLM (spatial lag)	SEM (spatial error)
Maternal characteristics			
Age (in years)	−0.004	−0.003	0.001
Education (above secondary)	−0.068**	−0.043	−0.001
Intended pregnancy	0.665***	0.546***	0.516***
Parity (3 + children)	−0.246***	−0.193***	−0.086*
Pregnancy outcome (adverse)	0.135***	0.124***	0.166***
Household & socio-economic			
Household head relation (head/spouse)	0.096***	0.106***	0.101***
Wealth (above rich)	0.03	0.009	0.064**
Caste (SC or ST)	−0.036**	−0.035***	−0.044***
Religion (minority)	−0.039***	−0.022*	−0.037**
Residence (urban)	0.01	0.019	−0.042**
Access & exposure			
Mass media exposure	0.244***	0.196***	0.232***
Health insurance coverage	0.051***	0.037***	0.051***
Health facility (public)	0.179***	0.177***	0.235***
(Constant)	0.138	0.005	0.031
Model fit statistics			
Observations (*N*)	707	707	707
Adjusted R-squared (*R*^2^)	0.61	0.66	0.75
Log likelihood	862.34	905.96	971.58
AIC	−1696.69	−1781.92	−1915.16
Spatial parameters			
Spatial lag (ρ)	—	0.257***	—
Spatial error (λ)	—	—	0.686***

*p*-value * < 0.05, ** < 0.01, *** < 0.001.

A positive association is observed between intended pregnancy and Q-ANC utilization, indicating that districts with higher proportions of planned pregnancies tend to report higher utilization rates. Similarly, the prevalence of adverse pregnancy outcomes is positively associated with Q-ANC utilization, suggesting that increased health risks may lead to greater engagement with antenatal care services.

In contrast, socio-cultural disadvantage appears to negatively influence Q-ANC utilization. The proportion of Scheduled Caste/Scheduled Tribe (SC/ST) populations and religious minority groups shows a negative association with Q-ANC utilization. Specifically, a one percentage point increase in the SC/ST population is associated with a decline of approximately 0.03 percentage points in Q-ANC utilization, while a similar increase in the proportion of religious minorities leads to a reduction of approximately 0.039 percentage points.

Access and exposure-related factors demonstrate a strong positive association with Q-ANC utilization. Districts with higher levels of mass media exposure show increased utilization, with a one percentage point increase in exposure associated with a 0.24 percentage point rise in Q-ANC utilization. Similarly, greater reliance on public healthcare facilities is associated with higher utilization rates, with a one percentage point increase in public healthcare use corresponding to a 0.18 percentage point increase in Q-ANC utilization.

Following the incorporation of spatial dependence, the overall model performance improved substantially, with the adjusted *R*^2^ increasing from 0.61 in the OLS model to 0.75 in the spatial regression models (Table 3). This improvement indicates that accounting for spatial effects provides a better explanation of the variation in Q-ANC utilization across districts.

**Table 3:** Comparison of OLS, SLM, SEM and GWR model performance.

Parameters	OLS	SLM	SEM	GWR
Adjusted *R*^2^	0.61	0.66	0.75	0.80
AICc	−1696.69	−1781.92	−1915.16	−2077.5
AICc reduction	—	85.23	218.47	380.81
RSS (residual sum of squares)	3.61	—	—	1.46

Source: Calculated by author.

Notably, the effect of household wealth becomes statistically significant in the spatial models, although it was insignificant in the OLS specification. Specifically, an increase of one percentage point in the proportion of individuals belonging to the richer or highest wealth quintile is associated with an increase of approximately 0.06 percentage points in Q-ANC utilization. This suggests that spatial dependence may have masked the true effect of economic status in the non-spatial model.

Furthermore, the reduction in Akaike Information Criterion (AIC) values observed in both the Spatial Lag Model (SLM) and Spatial Error Model (SEM), compared to the OLS model, indicates improved model fit. This decline in AIC values provides statistical evidence of the presence of spatial heterogeneity and justifies the application of spatial econometric approaches to better capture the underlying geographic processes influencing Q-ANC utilization.

### Determinants of Q-ANC: local model (GWR)

**Figure 3:**
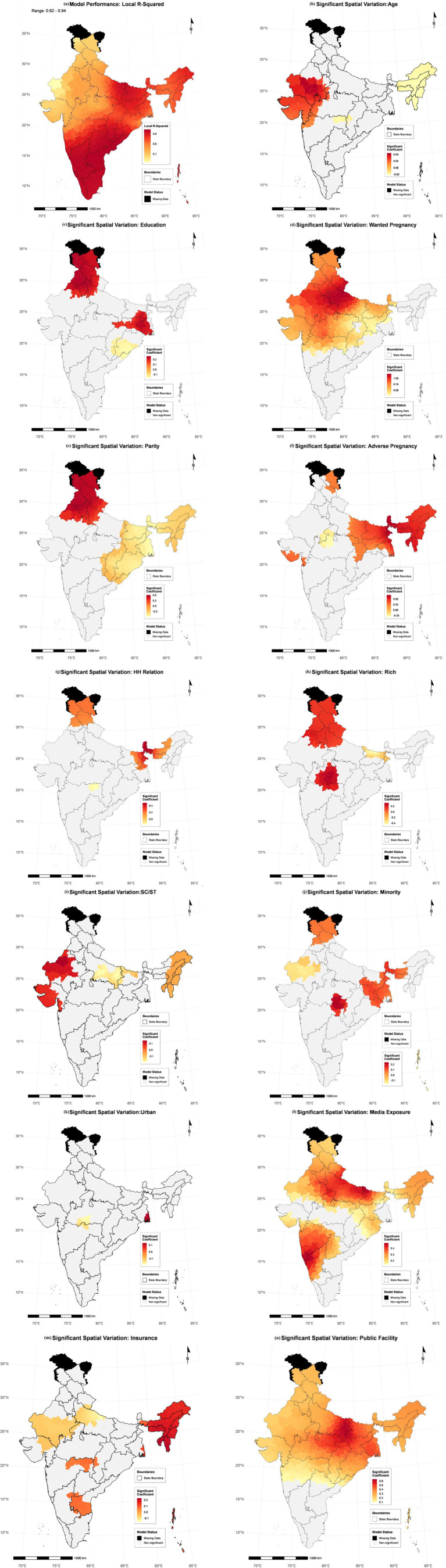
Local coefficients from GWR at the 5% significance level **(a)** Local **(b)** Mean age, **(c)** Education, **(d)** Intended pregnancy, **(e)** Parity, **(f)** Adverse pregnancy, **(g)** Household relation, **(h)** Wealth, **(i)** Social group, **(j)** Religion, **(k)** Residence, **(l)** Mass media, **(m)** Insurance, and **(n)** Public healthcare. Source: Computed by author.

The results of the geographically weighted regression (GWR) model indicate a substantial improvement in model performance after accounting for spatial heterogeneity in the relationships between predictors and Q-ANC utilization (Supplementary Table S4). The overall model fit improved considerably, with local *R*^2^ values ranging from 0.62 to 0.94, suggesting moderate to high explanatory power across districts (Figure 3a). The spatial distribution of local *R*^2^ values further reveals that model performance is relatively higher in districts of southern India, whereas comparatively moderate performance is observed across north western regions, indicating spatial variability in model fit.

The GWR results highlight significant spatial heterogeneity in the association between explanatory variables and Q-ANC utilization. A positive association between mean maternal age and Q-ANC utilization is observed in districts of Gujarat and Rajasthan, while a negative association is evident across several districts in northeastern India, suggesting region-specific demographic influences.

Intended pregnancy emerges as one of the strongest predictors of Q-ANC utilization, particularly across northern and western India (Figure 3d). The magnitude of association varies spatially, ranging from 0.50 to 1.0 percentage points. For instance, in districts of western Uttar Pradesh and Uttarakhand, a one percentage point increase in intended pregnancy is associated with nearly a one percentage point increase in Q-ANC utilization, indicating a strong localized effect. Similarly, adverse pregnancy outcomes show a predominantly positive association with Q-ANC utilization across eastern and northeastern regions, where increases of up to 0.50 percentage points are observed (Figure 3f). However, a few districts in north western Madhya Pradesh and eastern Rajasthan exhibit a negative association (approximately −0.25 percentage points), highlighting localized deviations from the overall trend.

Household-level characteristics also demonstrate considerable spatial variation. The proportion of women either household head or spouse relationship to the household head shows a strong positive association with Q-ANC utilization across districts in Bihar, West Bengal, Sikkim, and several northeastern states, with effect sizes reaching up to 0.40 percentage points (Figure 3g). In contrast, the effect of wealth status varies across regions (Figure 3h). While higher wealth concentration is positively associated with Q-ANC utilization in most districts, negative associations are observed in parts of Bihar and north western West Bengal, suggesting context-specific economic influences. The effect of social composition also varies spatially (Figure 3i). An increase in the proportion of Scheduled Caste/Scheduled Tribe (SC/ST) population is positively associated with Q-ANC utilization in districts of Rajasthan and Gujarat (approximately +0.10 percentage points), whereas negative associations of similar magnitude are observed in eastern Uttar Pradesh and northern Bihar.

Access and exposure-related factors exhibit consistently strong positive effects, although with varying magnitudes across regions. Mass media exposure is positively associated with Q-ANC utilization across most districts, with the strongest effects (up to +0.40 percentage points) observed in parts of Uttar Pradesh, Bihar, Maharashtra, Goa, and Karnataka (Figure 3l). Similarly, increased utilization of public healthcare facilities is associated with higher Q-ANC utilization across a majority of districts, with effect sizes ranging from 0.10 to 0.60 percentage points, particularly concentrated in eastern Uttar Pradesh and western Bihar (Figure 3n). In contrast, the effect of health insurance coverage demonstrates notable spatial heterogeneity (Figure 3m). Positive associations (up to +0.20 percentage points) are observed in districts of northeastern and southern India, whereas negative associations (around −0.10 percentage points) are evident in parts of western Uttar Pradesh, Rajasthan, and Gujarat.

Overall, the GWR findings underscore the presence of strong spatial non-stationarity in the determinants of Q-ANC utilization, indicating that both the direction and magnitude of associations vary significantly across regions. These results highlight the importance of localized contextual factors in shaping maternal healthcare utilization patterns across India.

## Discussion

The findings of the present study reveal substantial spatial variation in the utilization of quality antenatal care (Q-ANC) across districts of India. Districts located in southern India consistently demonstrate higher levels of Q-ANC utilization, whereas districts in Bihar, Uttar Pradesh, and the northeastern region exhibit comparatively lower utilization. A similar pattern is observed in the spatial clustering analysis, where hotspot regions are concentrated in southern India, while cold spot clusters are primarily located in Bihar, Uttar Pradesh, and northeastern states. These findings highlight a persistent regional divide in maternal healthcare utilization and indicate that improvements in service coverage remain uneven across geographic regions.

The improved performance of spatial models further suggests that the determinants of Q-ANC utilization are not spatially uniform but vary across geographic contexts. This spatial heterogeneity indicates that conventional non-spatial approaches may overlook important localized dynamics influencing maternal healthcare utilization. Therefore, incorporating spatial dimensions into the analysis provides a more nuanced understanding of regional disparities and strengthens the evidence base for context-specific policy interventions. Addressing such regional variations is essential not only for improving maternal health outcomes but also for advancing broader development goals related to health equity and social inclusion.

The spatial autocorrelation analysis confirms the presence of statistically significant clustering in Q-ANC utilization, reinforcing the existence of geographic inequality. The identification of hotspot and cold spot regions aligns with previous studies that have documented similar spatial patterns in maternal healthcare utilization across India (13, 38). These patterns may be attributed to underlying socio-economic and structural factors, including differences in educational attainment, household wealth, place of residence, and health system capacity (24, 39, 40). Furthermore, the observed north–south divide may reflect disparities in healthcare infrastructure, governance efficiency, and service delivery mechanisms, particularly in northeastern regions where access to healthcare services remains limited (13, 41).

A counterintuitive finding relates to educational attainment was observed in the study. Although the OLS model suggested a negative association between the proportion of women with secondary or higher education and Q-ANC utilization, this relationship was substantially attenuated and became statistically insignificant after accounting for spatial dependence. This pattern suggests that the initial association may have been influenced by spatial confounding and aggregation effects inherent in district-level analyses (42, 43). Educational attainment is often closely correlated with geographically clustered contextual characteristics such as household wealth, media exposure, urbanization, and healthcare accessibility (12). Consequently, the OLS coefficient may have captured broader spatial inequalities rather than an independent effect of education itself. The absence of a significant association in the spatial models indicates that educational attainment alone may not be a robust district-level predictor of Q-ANC utilization once spatial heterogeneity and contextual conditions are taken into account.

The results from spatial regression models indicate that factors such as intended pregnancy, adverse pregnancy outcomes, mass media exposure, health insurance coverage, and utilization of public healthcare facilities act as significant drivers of Q-ANC utilization, even after accounting for spatial dependence. The positive association between intended pregnancy and Q-ANC utilization suggests that women with planned pregnancies are more likely to initiate early antenatal care and adhere to recommended services, which is consistent with existing evidence (44–46). Similarly, the positive association between adverse pregnancy outcomes and Q-ANC utilization may reflect increased health-seeking behaviour among women with prior complications, although this relationship appears to vary across regions (47).

The GWR results further demonstrate substantial spatial non-stationarity in the determinants of Q-ANC utilization, indicating that both the direction and magnitude of associations differ across districts. For instance, the positive association between adverse pregnancy outcomes and Q-ANC utilization observed in eastern and northeastern regions contrasts with negative associations identified in parts of Madhya Pradesh and Rajasthan. While existing literature suggests that adverse experiences may reduce healthcare utilization due to fear or mistrust (48, 49), the observed positive associations in several regions may reflect the impact of targeted government interventions, such as the National Health Mission and Janani Suraksha Yojana (JSY), along with improvements in healthcare accessibility and awareness (50, 51).

The role of socio-economic and social stratification also exhibits considerable spatial variation. A positive association between Q-ANC utilization and the proportion of Scheduled Caste and Scheduled Tribe populations is observed in western India, whereas negative associations are evident in Bihar, Uttar Pradesh, and northeastern regions. This observed pattern may be attributed to policy interventions implemented under National Health Mission (NHM), such as Janani Suraksha Yojana (JSY) and Janani Shishu Suraksha Karyakram (JSSK). In addition, region-specific initiatives such as Mamta-Birth Waiting Home Scheme, which aim to improve healthcare access among marginalized populations in remote areas, may have contributed to better service utilization in western region (52, 53). In contrast, persistent barriers including social exclusion, discrimination, inadequate healthcare infrastructure, and severe geographical challenges continue to hinder healthcare utilization in regions such as Uttar Pradesh, Bihar, and parts of North-East India (54–57).

Similarly, household-level dynamics, such as the relationship of women to the household head, demonstrate region-specific effects that remain underexplored in the literature. The positive association observed in northern and north western regions, contrasted with negative patterns in eastern and northeastern regions, suggests that local socio-cultural norms and intra-household decision-making processes may play an important role in shaping maternal healthcare utilization (58, 59).

The study also identifies significant spatial variation in the effect of health insurance coverage on Q-ANC utilization. While positive associations were observed in the northeastern and southern regions, negative associations were found in parts of western India and Uttar Pradesh. The positive association between health insurance and maternal healthcare utilization is consistent with findings from existing national-level studies (60, 61), whereas the negative regional associations appear counterintuitive to the documented evidence. A national-level study from Ghana reported that health insurance was negatively associated with institutional delivery due to institutional mechanism failures and the continued demand for out-of-pocket payments even among insured individuals (62). These findings highlight the need for further investigation into region-specific mechanisms underlying the negative association between health insurance coverage and Q-ANC utilization (63).

In contrast, access and exposure-related factors, particularly mass media exposure and utilization of public healthcare facilities, demonstrate consistently positive associations with Q-ANC utilization across most regions. These findings are consistent with previous studies indicating that improved access to public healthcare infrastructure and increased health awareness through media exposure contribute significantly to maternal healthcare utilization and improved health outcomes (24, 64–66). However, the magnitude of these effects varies spatially, further reinforcing the importance of localized contextual factors.

Overall, the findings of the present study contribute to the existing literature by highlighting the importance of spatial heterogeneity in understanding maternal healthcare utilization. By demonstrating that the determinants of Q-ANC utilization vary across regions, the study underscores the limitations of uniform policy approaches and emphasizes the need for geographically targeted interventions. Considering local socio-economic and health system condition may enhance the effectiveness of maternal healthcare programs and contribute to achieving Sustainable Development Goals related to health (SDG-3), gender equality (SDG-5), and reduced inequalities (SDG-10).

At the same time, the study identifies several areas requiring further investigation. Some findings, particularly the region-specific contradictory effects of variables such as adverse pregnancy outcomes, health insurance coverage, and wealth status, deviate from established literature. These inconsistencies highlight the need for more localized and mixed-method research to better understand the contextual factors driving such variations. Future studies should explore these dynamics in greater depth to inform more effective and context-sensitive policy interventions.

## Strengths and limitations

The present study has several important strengths. First, it addresses a critical public health issue by examining spatial inequalities in quality antenatal care (Q-ANC) utilization across districts of India, providing evidence relevant to maternal health improvement and the achievement of Sustainable Development Goals. Second, the analysis utilizes nationally representative NFHS-5 data, ensuring broad geographic coverage and policy relevance. Third, the integration of multiple spatial analytical approaches, including Global and Local Moran’s I, spatial econometric models (SLM and SEM), and geographically weighted regression (GWR), enables a comprehensive assessment of both global and local spatial processes influencing Q-ANC utilization. Finally, by focusing on the content of antenatal care rather than solely the number of ANC visits, the study provides a more comprehensive assessment of service quality during pregnancy.

Despite these strengths, several limitations should be acknowledged. First, the study adopts an ecological design based on district-level aggregated indicators; therefore, the observed associations should not be interpreted at the individual level. Second, the cross-sectional nature of NFHS-5 data limits the ability to establish temporal relationships or causal inferences. Third, although the operationalization of Q-ANC as receipt of at least five out of seven recommended ANC components is consistent with previous DHS/NFHS-based studies, alternative definitions may yield somewhat different estimates of coverage. Finally, while GWR is useful for identifying spatial heterogeneity and localized associations, the resulting coefficients should be interpreted as exploratory and context-specific rather than causal estimates, as local parameter estimates may be sensitive to bandwidth selection and spatial scale.

## Conclusion

The present study extends the conventional understanding of maternal healthcare utilization by incorporating spatial analytical techniques to examine geographic variation in quality antenatal care (Q-ANC) across districts of India. The findings reveal pronounced spatial disparities, with clear regional clustering in both high- and low-performing areas, indicating that Q-ANC utilization is not randomly distributed but shaped by underlying socio-economic and health system factors. Key determinants such as adverse pregnancy outcomes, mass media exposure, health insurance coverage, and utilization of public healthcare facilities are found to be significantly associated with Q-ANC utilization. However, the geographically weighted regression results demonstrate substantial spatial non-stationarity, with both the magnitude and direction of these associations varying across regions. This highlights that aggregate-level relationships may conceal important localized dynamics and reinforces the need to interpret determinants within their specific geographic context.

From a policy perspective, these findings underscore the limitations of uniform approaches to maternal healthcare interventions in a geographically diverse country like India. The effectiveness of policies is contingent upon regional socio-economic conditions, healthcare infrastructure, and access to information, necessitating the adoption of context-specific and geographically targeted strategies. Strengthening public healthcare systems, improving the reach and effectiveness of health communication, and ensuring equitable implementation of health insurance schemes are critical for enhancing Q-ANC utilization, particularly in low-performing districts of northern and northeastern India. By integrating spatial perspectives into policy design and implementation, it is possible to address persistent regional inequalities more effectively and accelerate progress toward improved maternal and child health outcomes, as well as broader goals related to health equity and social development.

## Data Availability

The datasets presented in this study can be found in online repositories. The names of the repository/repositories and accession number(s) can be found at: https://dhsprogram.com/data/.
