# Supplementary material for: Spatial inequalities in quality antenatal care in India: a district-level analysis
Source: Front Public Health. 2026 Jul 17;14:1878310. doi: 10.3389/fpubh.2026.1878310 (PMC13424245; doi:10.3389/fpubh.2026.1878310)
Supplement: Supplementary file 1 [file Table_1.DOCX]

Supplementary table

Table S1: Monte Carlo test for spatial variability of GWR coefficients

| **Variables** | **p-value** | **Spatial Variability** |
| --- | --- | --- |
| Intercept | 0 | Significant |
| Mean Age | 0.001 | Significant |
| Education (Secondary+) | 0.061 | Not Significant |
| Wealth (Rich+) | 0 | Significant |
| SC/ST Population | 0.006 | Significant |
| Minority Population | 0.011 | Significant |
| Urban Residence | 0.363 | Not Significant |
| Intended Pregnancy | 0.001 | Significant |
| Mass Media Exposure | 0.142 | Not Significant |
| Public Health Facility Use | 0 | Significant |
| High Parity | 0 | Significant |
| Adverse Pregnancy Outcome | 0.001 | Significant |
| Health Insurance Coverage | 0 | Significant |
| Household Relation | 0.009 | Significant |

Table S2: Variance inflation factor (VIF) for explanatory variables

| **Variables** | **Variance inflation factor value** |
| --- | --- |
| Mean Age | 2.1 |
| Education (Secondary+) | 3.71 |
| Wealth (Rich+) | 4.41 |
| SC/ST Population | 1.6 |
| Minority Population | 2.07 |
| Urban Residence | 2.45 |
| Intended Pregnancy | 1.28 |
| Mass Media Exposure | 3.91 |
| Public Health Facility Use | 1.43 |
| High Parity | 3.19 |
| Adverse Pregnancy Outcome | 1.49 |
| Health Insurance Coverage | 1.25 |
| Household Relation | 2.06 |

Table S3: Description of district-level variables used in the study

| **Variable** | **Description** |
| --- | --- |
| Age (years) | Mean age of respondents at the district level |
| Education (Above Secondary) | Percentage of individuals with secondary or higher education |
| Intended Pregnancy | Percentage of individuals reporting their pregnancy as intended |
| Parity (3+ Children) | Percentage of individuals who have given birth to three or more children |
| Pregnancy Outcome (Adverse) | Percentage of individuals with a history of adverse pregnancy outcomes |
| Household Decision Maker (Head/Spouse) | Percentage of individuals who are either the household head or the spouse of the head |
| Wealth (Above Rich) | Percentage of individuals belonging to the rich and richest wealth quintiles |
| Caste (SC/ST) | Percentage of individuals belonging to Scheduled Castes or Scheduled Tribes |
| Religion (Minority) | Percentage of individuals belonging to non-Hindu religious groups |
| Residence (Urban) | Percentage of individuals residing in urban areas |
| Mass Media Exposure | Percentage of individuals exposed to mass media (newspapers, radio, or television) |
| Health Insurance Coverage | Percentage of individuals covered by any health insurance scheme |
| Health Facility (Public) | Percentage of individuals utilizing public healthcare facilities for ANC visits |

Table S4 Descriptive statistics of the explanatory variables of the geographically weighted regression analysis

| **Variables** | **Min** | **1st Qu.** | **Median** | **3rd Qu.** | **Max** |
| --- | --- | --- | --- | --- | --- |
| Age (in Years) | -0.02 | -0.01 | 0.00 | 0.00 | 0.04 |
| Education (Above Secondary) | -0.17 | -0.03 | 0.06 | 0.13 | 0.27 |
| Intended pregnancy | -0.12 | 0.21 | 0.44 | 0.78 | 1.19 |
| Parity (3+ Children) | -0.58 | -0.33 | 0.02 | 0.14 | 0.61 |
| Pregnancy outcome (Adverse) | -0.29 | -0.05 | 0.16 | 0.32 | 0.69 |
| Household head relation (Head/Spouse) | -0.16 | -0.03 | 0.02 | 0.10 | 0.48 |
| Wealth (Above Rich) | -0.45 | -0.01 | 0.04 | 0.11 | 0.32 |
| Caste (SC or ST) | -0.19 | -0.05 | 0.02 | 0.04 | 0.15 |
| Religion (Minority) | -0.17 | -0.01 | 0.03 | 0.06 | 0.24 |
| Residence (Urban) | -0.19 | -0.02 | -0.01 | 0.04 | 0.13 |
| Mass media exposure | -0.04 | 0.16 | 0.24 | 0.31 | 0.49 |
| Health Insurance Coverage | -0.15 | -0.03 | 0.03 | 0.06 | 0.23 |
| Health Facility (Public) | -0.03 | 0.20 | 0.28 | 0.35 | 0.64 |
| Adjusted R^2 | 0.80 |  |  |  |  |
| AICc | -2077.52 |  |  |  |  |
| Best Bandwidth | 168 |  |  |  |  |
